# Attenuation of Photoelectron Emission by a Single Organic Layer

**DOI:** 10.1021/acsami.2c02996

**Published:** 2022-05-10

**Authors:** Thorsten Wagner, Grażyna Antczak, Michael Györök, Agata Sabik, Anna Volokitina, Franciszek Gołek, Peter Zeppenfeld

**Affiliations:** † Johannes Kepler University, Institute of Experimental Physics, Surface Science Division, Altenberger Strasse 69, 4040 Linz, Austria; ‡ University of Wroclaw, Institute of Experimental Physics, Pl. M. Borna 9, 50-204 Wroclaw, Poland

**Keywords:** cobalt-phthalocyanine, work function, photoelectron emission microscopy, Anderson method, Ag(100), Fowler−DuBridge theory, attenuation of electrons, inelastic mean free path

## Abstract

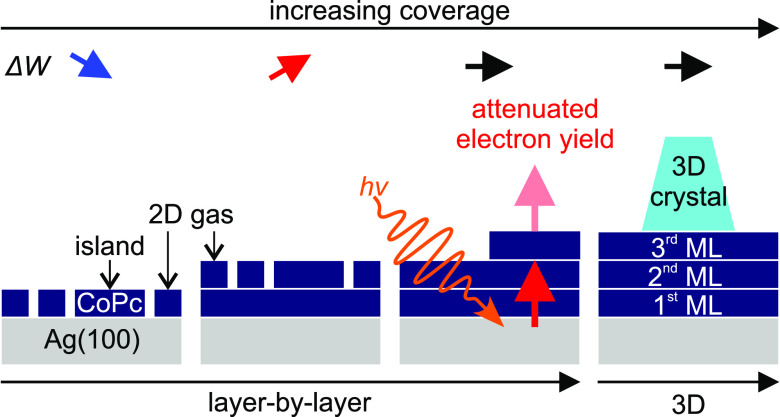

We report an in situ study of the thin-film growth of cobalt-phthalocyanine on Ag(100) surfaces using photoelectron emission microscopy (PEEM) and the Anderson method. Based on the Fowler–DuBridge theory, we were able to correlate the evolution of the mean electron yield acquired with PEEM for coverages up to two molecular layers of cobalt-phthalocyanine to the global work function changes measured with the Anderson method. For coverages above two monolayers, the transients measured with the Anderson method and those obtained with PEEM show different trends. We exploit this discrepancy to determine the inelastic mean free path of the low-energy electrons while passing through the third layer of CoPc.

## Introduction

Organic thin films are successfully applied as functional layers in many electronic devices. The orientation of the molecules, which form the active films, the film morphology, and its crystallinity affect the performance of such devices.^1,2^ Therefore, the correlation between electronic and structural properties of a system and their evolution during deposition are particularly important for controlling the performance of such organic thin films. A crucial parameter in this context is the work function of the system. The organic–metal interactions lead to a charge redistribution at the interface or even perhaps to a charge transfer between the molecule and the substrate. Both effects induce changes in the work function.^3^ For molecules in the second layer and above, the molecule–molecule interactions are more pronounced. Therefore, the stuctural and electronic properties evolve rapidly within the initial layers until they reach bulk properties.

**Figure 1:**
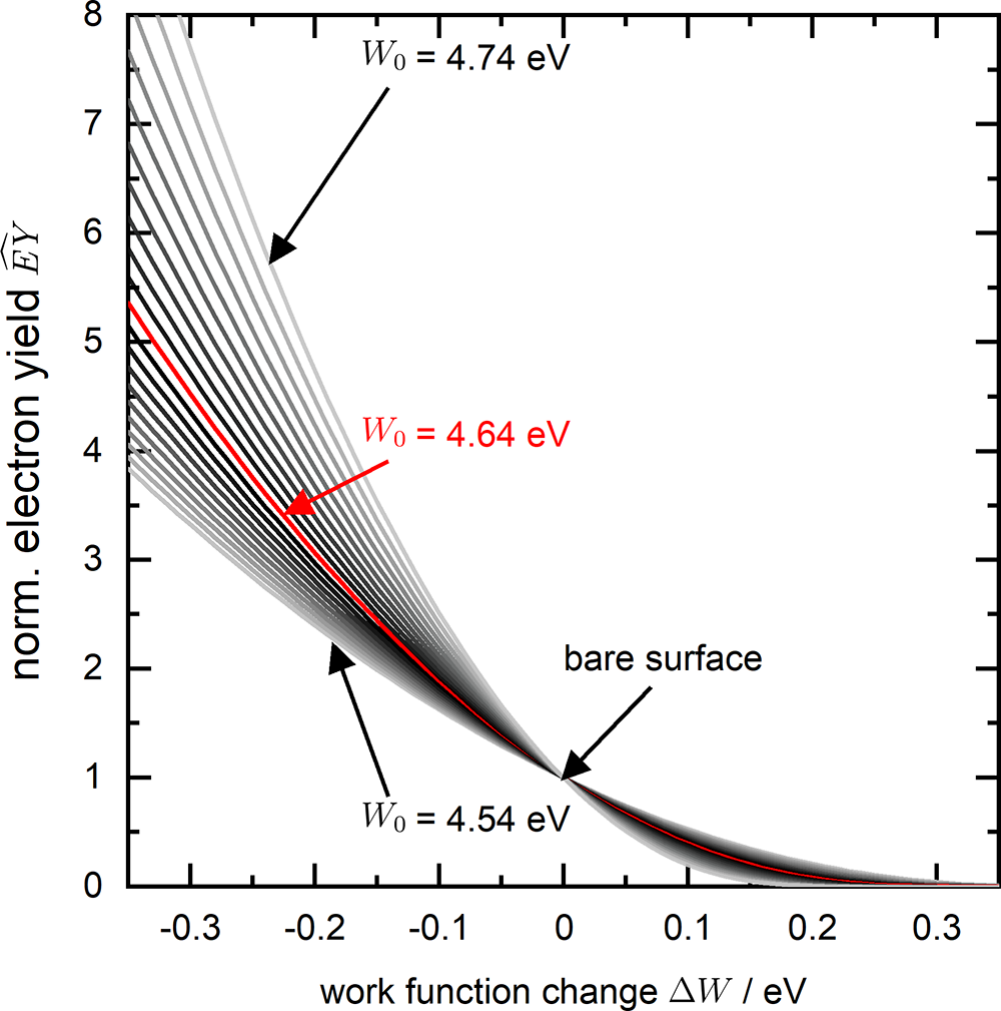
Normalized electron yield  as a function of work function change Δ*W* based on eq 2 for a surface temperature of 300 K. The exact line shape depends on the initial work function *W*_0_ of the bare Ag(100) surface. In the present calculations, *W*_0_ was varied in steps of 0.01 eV between 4.54 eV and 4.74 eV, while the photon energy was fixed at 4.9 eV.

**Figure 2:**
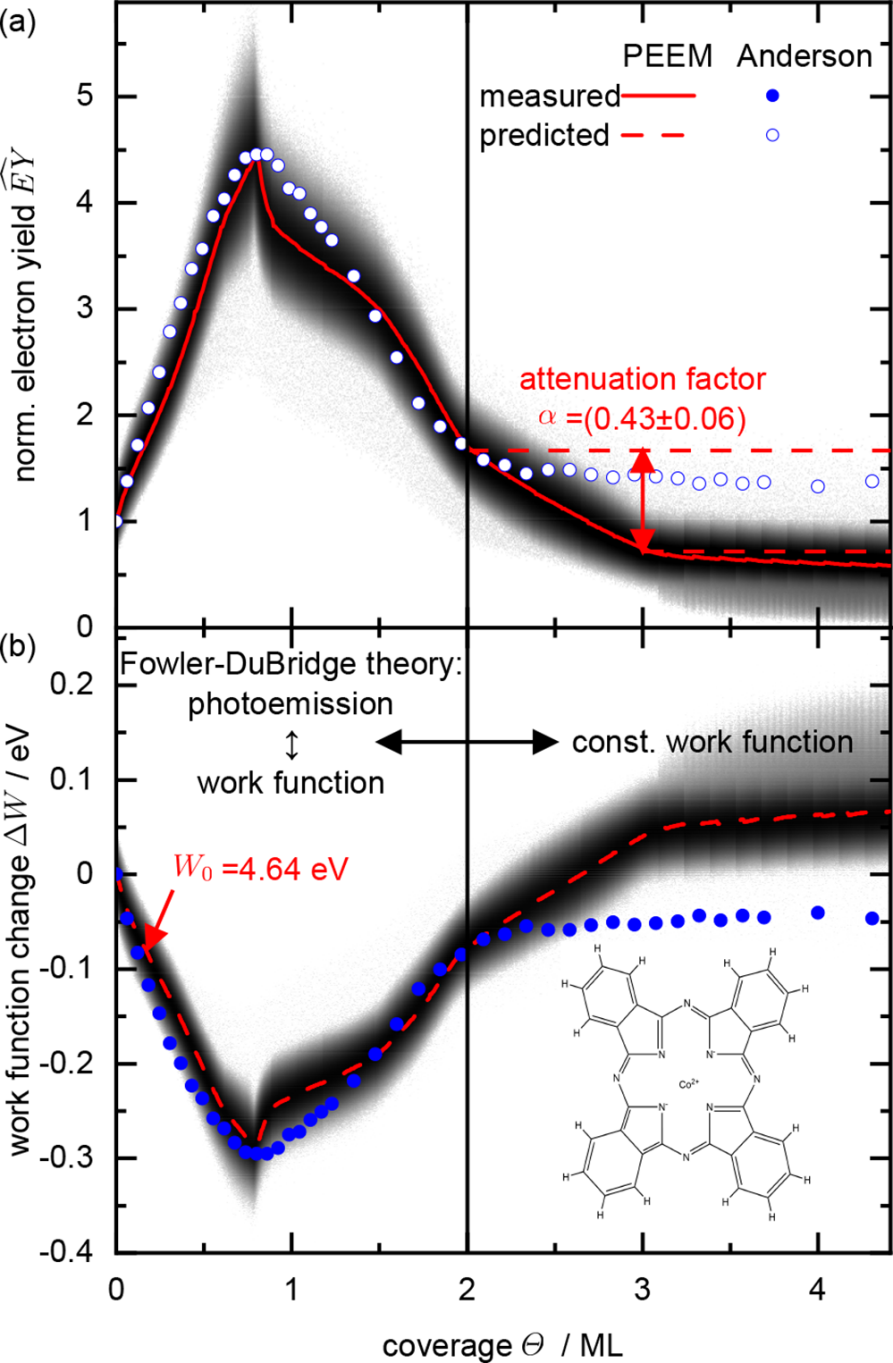
(a) Evolution of the normalized electron yield measured with photoelectron emission microscopy (PEEM) during the deposition of CoPc on a Ag(100) surface kept at room temperature. The mean electron yield MEY is shown as the red solid line, while the distribution of the electron yield within each image is presented as a histogram in gray scale in the background. (b) Transient of Δ*W* measured by the Anderson method (filled blue circles) recorded independently from (a). In both plots, eq 2 was used to extract the values of  based on Δ*W* ((a), open blue circles) or Δ*W* based on the  ((b), dashed red line), respectively. The false color background in (b) shows the (calculated) distribution of Δ*W* obtained from the pixel-wise evaluation of the PEEM data. The inset in (b) shows the structural formula of CoPc.

As a model system for a metal–organic interface, we selected ultrathin films of cobalt-phthalocyanine (CoPc; see the inset in Figure 2b for the structural formula) adsorbed on Ag(100) surfaces. Phthalocyanines are nearly planar and widely studied organic molecules due to their promising properties for solar cells,^4^ field-effect transistors,^5,6^ sensors,^7^ and light-emitting devices.^8^ A Stranski–Krastanov growth mode is often observed during the deposition of such molecular films. In general, the transition from two-dimensional (2D) growth (layer-by-layer) to a three-dimensional (3D) one (crystallites, needles, or whiskers) can be accompanied by a change from flat-lying to upright standing molecules. This has, of course, a strong impact on the electronic signature of the resulting films.^9,10^

In this study, we employed two techniques: photoelectron emission microscopy (PEEM) and the Anderson method (see the Supporting Information and refs (11, 12) for further details). Both can be applied in situ during molecular growth and provide informationabout the sample with comparable temporal resolution. PEEM allows imaging the local electron yield (EY) with a field of view ranging from 10  to several 100 . The image contrast is affected by the work function as well as the accessible density of states. The energy and polarization of the photons used for excitation^13^ and the sample morphology (due to shadowing) also play a role here. If the photoelectrons are not excited in the top layer of the surface, electron transport must be considered, too.^14−17^ The transients of the (local and mean) electron yield (EY) extracted from PEEM movies recorded during deposition of organic molecules on the surface can thus be related partly, but not solely to changes in work function. Therefore, it is, in general, difficult to derive a quantitative value for the work function based on the intensity of a PEEM image, alone.

On the other hand, the Anderson method allows direct measurements of changes in work function (Δ*W*) on a macroscopic scale during growth. As a second parameter, the reflectivity of low-energy electrons (*R* for *E*_kin_ in the range of 0 eV–5 eV) can be extracted from the data.

## Results and Discussion

### Fowler–DuBridge Theory

In the following, we use the evolution of the intensities in PEEM images to estimate the work function change upon adsorption of the cobalt-phthalocyanine molecules on Ag(100) surfaces and compare this to Δ*W*(Θ) obtained with the Anderson method. The results of the two experiments are linked via the Fowler–DuBridge theory.^18−20^ The technical details of both used techniques and the raw data are presented in the Supporting Information. According to the Fowler–DuBridge theory,^18−20^ the electron yield is proportional to the current density *J* induced by illuminating the sample with photons of energy *h*ν
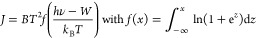
1where the parameter *B* depends on the photon flux and the geometrical design of the PEEM,^21^*T* denotes the thermodynamic temperature, and *k*_B_ is the Boltzmann constant. During a deposition experiment, it is safe to assume that *B* and *T* do not change. Furthermore, the Fowler–DuBridge theory is only valid if the sample is illuminated with photons, which excite electrons near the Fermi level.^22^

It is convenient to determine the normalized electron yield . It can be achieved by dividing eq 1 by the value EY_0_ determined for the initial growth state.
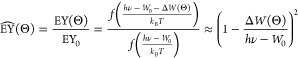
2where *W*_0_ denotes the initial work function.

The last expression represents a series expansion that is valid for low temperatures (neglecting higher-order terms).^21^Equation 2 can be applied to corresponding pixels with coordinates (*x*, *y*) of a series of *N* images acquired during the growth of an ultrathin organic film. In this case, EY_0_(*x*, *y*) is the electron yield of the pixel at coordinates (*x*, *y*) when the shutter is first opened. We also use the mean values of the electron yield (MEY) averaged over the field of view of 130 . Assuming a constant flux of CoPc molecules, these transient parameters can be expressed as a function of coverage Θ.

Equation 2 is used to predict the normalized mean electron yield () based on the relative change in the work function (Δ*W*) measured with the Anderson method during deposition. In turn, the inversion of eq 2 allows us to predict the changes in the work function (Δ*W*(Θ)) based on the normalized mean electron yield measured by PEEM. The precise relation between  and Δ*W* is affected by (i) the energy *h*ν of the incident photons and (ii) the absolute initial work function *W*_0_ of the bare silver substrate (see discussion of Figure 1 below).

For the PEEM experiments discussed here, a Hg lamp was used, whose spectral distribution is shown in ref (23). Assuming that the photoelectrons originate from the silver surface, only photons with an energy above *W*_0_ contribute to the photoemission process. In this energy range, the Hg lamp has a strong spectral line with a photon energy of 4.9 eV. Therefore, we assume that the light of the Hg lamp can be considered monochromatic with an energy of 4.9 eV.

Unfortunately, the Anderson method does not provide information on the absolute value of *W*_0_, but only on the incremental changes Δ*W*, i.e., during stepwise deposition of molecules. For the pristine Ag(100) surface, the value of *W*_0_ may vary from preparation to preparation due to impurities on the surface or structural defects such as steps or step bunches. Although different methods were used to determine an absolute value of the work function, an uncertainty in the order of 0.2 eV remains.^24^ As the basis for the further discussion, we take the value *W*_0_ = 4.64 eV given in ref (25).

Figure 1 shows the sensitivity of  to the absolute value of *W*_0_, when the sample is illuminated with photons of energy *h*ν = 4.9 eV. Eq 2 is plotted here for values of *W*_0_ between 4.54 eV and 4.74 eV. The curvature of the curve  depends strongly on the value of the selected *W*_0_.

### Initial Layers

**Figure 3:**
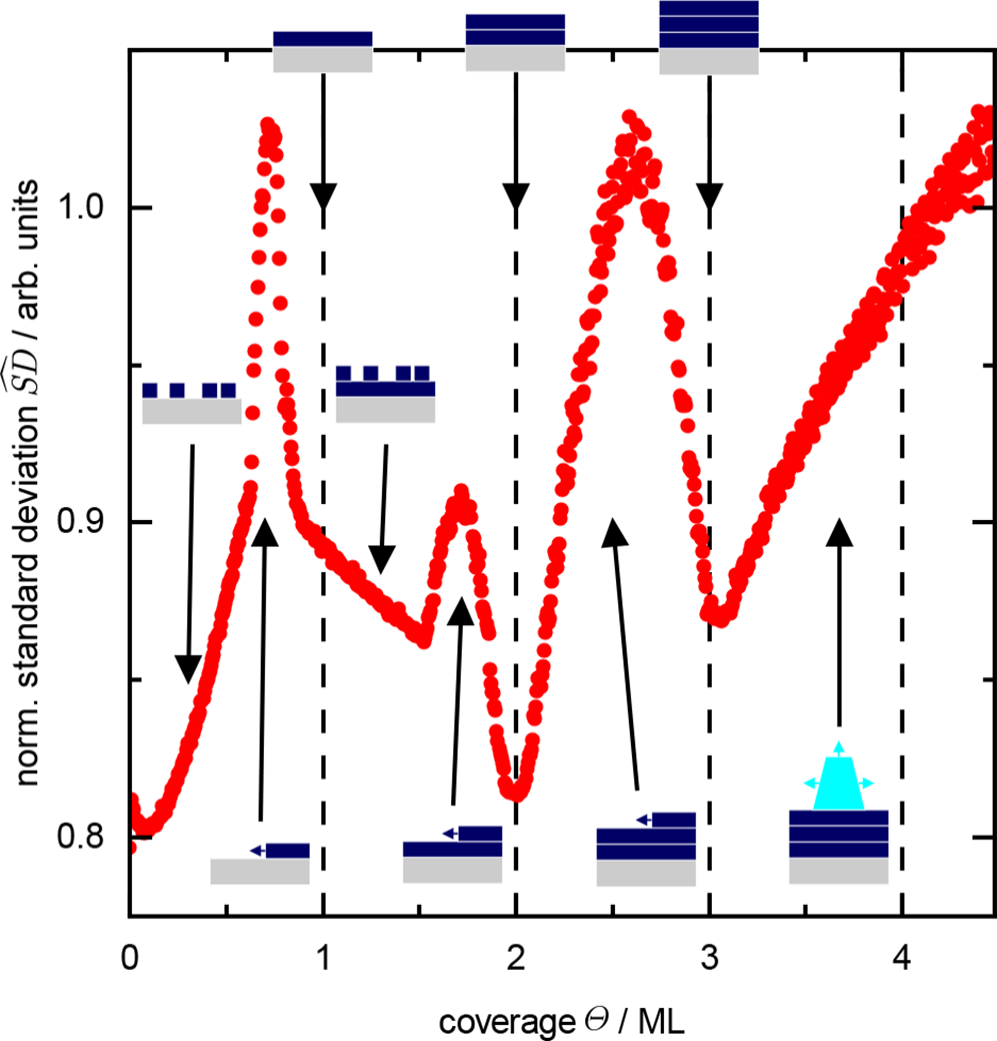
Normalized standard deviation () evaluated from the electron yield of the PEEM images obtained during CoPc deposition on Ag(100).

To match the experiments performed independently, we had to correlate the actual (accumulated) deposition time with the stages of growth. According to eq 2, a decrease in the work function *W*, i.e., Δ*W* < 0, as measured with the Anderson method (see filled blue circles in Figure 2b), should coincide with an increase in MEY (see the red solid line in Figure 2a) recorded in the PEEM, and vice versa. It is clear that the minimum of Δ*W*(Θ) and the maximum of  are associated with the same stage of growth, thus providing the link between the two data sets. PEEM can be used to identify different growth stages by analyzing the evolution of the normalized standard deviation () during the deposition. The details of the procedure are discussed in ref (26). From the plot of  in Figure 3, it can be seen that the maximum of  is at a coverage of 0.8 ML. Therefore, the minimum of Δ*W* obtained from the Anderson method should also correspond to a coverage of 0.8 ML. Note that in the previous work of Sabik et al.,^11,12^ the minimum of Δ*W* was assigned to a full monolayer (1 ML) of CoPc, whereas with the revised definition, we find that 1 ML corresponds to the minimum of the low-energy electron reflectivity, *R*(Θ) (see Figure S1 in the Supporting Information).

Based on this definition of a monolayer, we can interpret the transient Δ*W*(Θ) measured with the Anderson method as follows: the linear decrease of the work function indicates that each molecule initially affects the work function *W* of the system by the same constant amount. This means that the surface dipoles induced by the individual CoPc molecules interact only weakly on the surface. This is confirmed by ref (27), which reports a weak repulsive interaction between individual molecules. Such repulsive lateral interaction can give rise to a molecular 2D gas, in which individual molecules move freely on the surface. Depending on the density of such a gas phase, the molecules maintain (on average) a certain distance between each other but do not crystallize into larger islands.^28,29^ The analysis of the PEEM images presented below confirms the absence of a 2D condensation on a scale larger than the resolution limit of the PEEM (here about 150 nm). The molecules form either a pure molecular 2D gas phase or a mixed phase, in which smaller aggregates (clusters of molecules) are in equilibrium with a 2D gas phase.^30^ Above a coverage of 0.6 ML, the data obtained with the Anderson method show a deviation from the linear decrease of Δ*W*. This can be explained by depolarization caused by interactions between individual CoPc dipoles, which is known as the “Topping effect”.^31^ The global minimum of the work function is reached for 0.8 ML. At this point, the slope of the PEEM transient undergoes a rather abrupt change in sign from a positive to a negative one. This could be evidence for a structural transformation. The predominant structure reported for a monolayer of CoPc on Ag(100) is a (5 × 5)R0° superstructure. However, other local structures have been reported: a rotated (5 × 5)R37° phase, a less dense R11° phase, and a denser (7 × 7)R0° one.^32−34^ At this stage of deposition, some interlayer transport between the first and second layers might also take place.

Between 0.8 ML and the completion of the second monolayer, the Anderson method yields an almost linear increase in the work function with increasing coverage. At a coverage around 2.0 ML, the work function suddenly levels off and remains constant for thicker films. The final value lies 0.05 eV below the initial value of the bare surface. We explain the saturation of the work function with the negligible interaction between the substrate and the molecules in the third and higher layers. The PEEM data show later that the wetting layer is closed after deposition of 3.0 ML. When more molecules are deposited, 3D crystallites are formed on top of the wetting layer. The electronic properties of such crystallites should already be very close to those of the bulk crystal. In contrast to the deposition of CuPc on Ag(100),^35^ the work function after deposition of the second CoPc layer almost recovers to the initial value of the bare surface. Here, we can only speculate about possible reasons: (i) the molecules of the second layer induce a dipole facing in the opposite direction to that of the first layer or (ii) when the molecules are deposited in the second layer, the underlying first layer is altered in its electron distribution and/or structural arrangement.

Figure 2a shows the changes in  as a sequence of histograms (evaluated over all pixels of an image) in the background together with the mean values  indicated by the red line in front. In particular, the histograms illustrate the spreading of the data within each image. Since the absolute intensity also affects the standard deviation, Figure 3 shows the normalized standard deviation, . The EY(Θ) data in Figure 2a were converted into corresponding work function changes Δ*W*(Θ) using the approximation in eq 2 with *W*_0_ = 4.64 eV for the work function of the bare Ag(100) surface. The results are shown in Figure 2b via histograms in the background and mean values (dashed red line) in the front.

**Figure 4:**
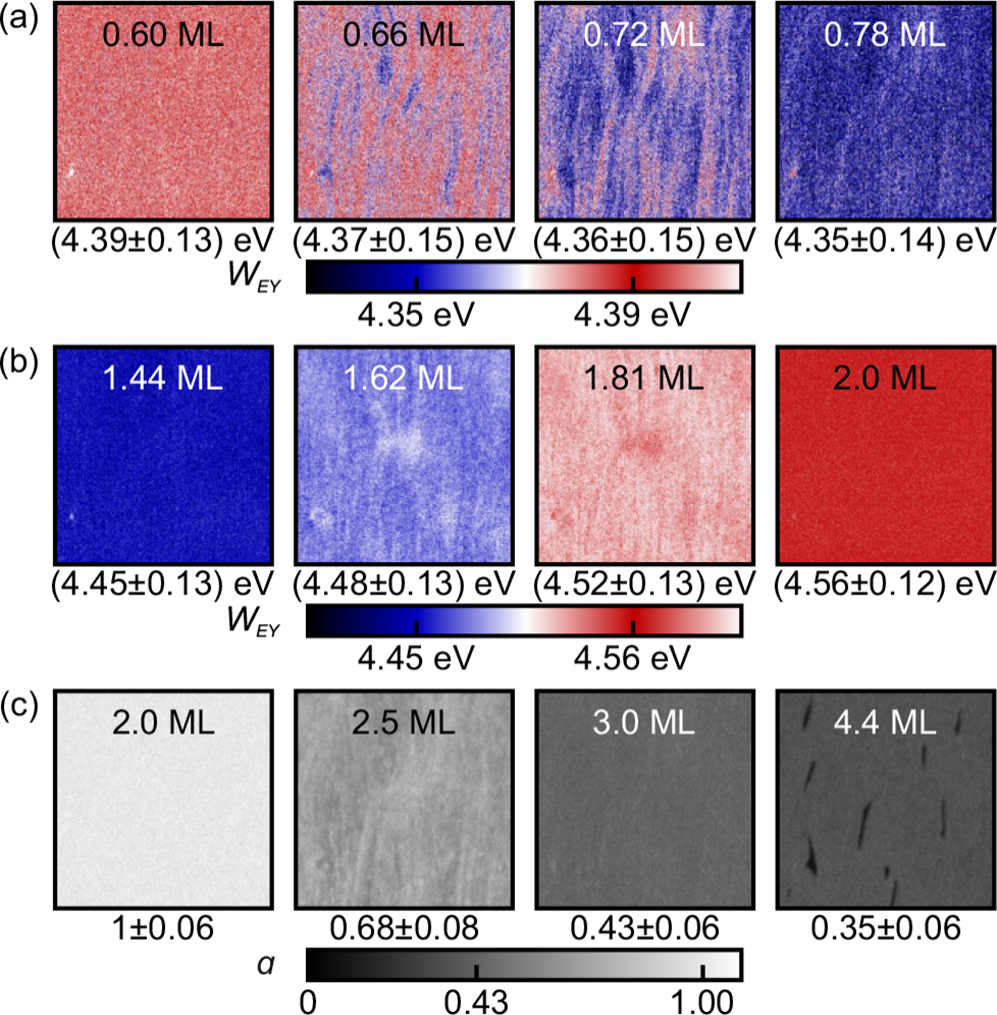
(a, b) Selected work function images calculated from the PEEM data using the approximation of the Fowler–DuBridge relation in eq 2 with *W*_0_ = 4.64 eV and *hν* = 4.9 eV. All images show the same position on the sample with an area of 50 by 50 . (c) Normalized electron yield α calculated according to eq 4. The reference, EY(2 ML), was obtained from the pixel-based average of three images at a coverage of Θ = 2.0 ML.

Up to a CoPc coverage of about 0.6 ML, there is an almost linear increase of , corresponding to an increasing brightness of the images, while the sample morphology does not change. The increase of the intensity combined with the lateral uniformity of the PEEM images can be related to the presence of CoPc molecules in a structurally homogeneous phase, namely, a 2D gas phase. Between 0.6 ML and 0.8 ML, the slope of the  transient slightly decreases compared to the initial situation. In this coverage range, the corresponding  transient also increases sharply (see Figure 3). We assume that the 2D gas phase reaches a critical density at Θ ≈ 0.6 ML and that 2D condensation sets in. In the PEEM images, a condensed phase appears with a higher electron emission than the coexisting gas phase. Between 0.6 ML and 0.8 ML, the regions with higher emission expand at the expense of regions with lower emission (see Figure 4). Finally, at a coverage of 0.8 ML,  reaches its maximum and the mean electron yield is 4.5 times higher than the initial value (MEY_0_) of the bare silver surface. At the same time, the work function *W* (see Figure 2b) reaches its minimum of about 0.30 eV below the initial *W*_0_ of the clean Ag(100) surface.

In general, the PEEM images in this coverage range can be interpreted as a direct mapping of the work function. Therefore, we use the inversion of eq 2 to estimate the local variation of the work function based on the PEEM images according to
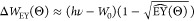
3In addition to the measured , we used *h*ν = 4.9 eV for the photon energy and *W*_0_ = 4.64 eV as the work function of the bare silver surface.^25^ Selected images for coverages between 0.6 ML and 0.8 ML are shown in Figure 4a. At a coverage of Θ = 0.6 ML, the mean work function is 4.39 eV. It decreases to 4.35 eV when the coverage reaches about 0.8 ML. As revealed by the pattern formation in the coverage range between 0.6 ML and 0.8 ML in Figure 4a, this transition proceeds locally via the switching from *W* = 4.39 eV–4.35 eV within certain regions, which expand and finally coalesce for Θ = 0.8 ML.

A rapid decrease in  is observed for coverages between 0.8 ML and 1.0 ML accompanied with a sharp drop of the normalized standard deviation  (see Figure 3) correlated with the disappearance of almost all lateral heterogeneities of the electron yield in the PEEM images. This behavior can be associated with the completion of the first molecular layer and rearrangements within it.

After deposition of an equivalent between 1.0 ML and about 1.5 ML of CoPc, we observe a linear decrease of . The analysis of  again shows that a phase is formed, in which the structures are smaller than the lateral resolution of PEEM in use (see Figure 3). The series of PEEM images in this stage reveals a uniform decrease in the electron yield. The critical coverage for the transition to a condensed phase (with structures larger than the resolution limit of the PEEM given here by the lateral pixel size of about 150 nm) is reached at a coverage of about 1.5 ML. At this point, the slope of  changes, while  develops another sharp peak. As with the first layer, this behavior is indicative of a 2D condensation transition, but this time in the second layer. As before, this transition is also reflected in the PEEM images, which reveal the coexistence of two emission states on the surface—see Figure 4b. With increasing CoPc coverage, the regions with low emission grow at the expense of those with high emission. Using eq 3, we estimate the work function at 1.5 ML to be 4.45 eV. The PEEM image obtains its (full) lateral homogeneity at 2.0 ML when the second layer is completely filled. The image corresponds to a mean work function of 4.56 eV. The pattern formation at intermediate coverages is accompanied by a steady decrease of the mean electron yield corresponding to an increase in the work function, as shown in Figure 4b. It is very likely that the larger regions, where the condensation sets in, initially correspond to decorated step bunches on the Ag(100) surface but that nucleation also takes place on the terraces in between. However, small nucleation centers up to several nanometers in diameter are not visible in the PEEM due to its limited spatial resolution.

The measured and calculated transients for the work function *W*(Θ) shown in Figure 2 should be identical in shape if the Fowler–DuBridge theory would be applicable over the entire coverage range. Some rescaling of the values could be achieved by varying the value of *W*_0_ of the bare silver surface, which is not precisely known. In the Supporting Information, the conversions for other *W*_0_ values in analogy to Figure 2 are shown. The best agreement is obtained for *W*_0_ = 4.64 eV. In general, there is a fairly good overlap between the curves up to a coverage of 2.0 ML. For higher coverages, however, the PEEM data suggest a further decrease of the work function, whereas the Anderson method yields a saturation at around 2 ML of CoPc.

The remaining differences between the transients shown in Figure 2 for coverages up to 2 ML can be related to the local versus macroscopic origin of the photo-emitted electrons in the two methods, respectively: for the Anderson method, the electrons directed onto the surface from far away will always target the areas with the lowest work function. On the other hand, the photoemission observed in PEEM is a local process^36−38^ so that the data points correspond to the full range of local work functions on a surface: step edges vs terraces but also different thicknesses of the CoPc thin film on the surface. Accordingly, values for the work function measured with the Anderson method should rather lie at the lower edge of the distribution of the work functions derived from the PEEM data (shown as the gray background in Figure 2b).

### Attenuation due to the 3rd Layer

In the PEEM experiments, we find that the layer-by-layer growth continues for the third monolayer:  decreases linearly with no relevant change in slope—see Figure 2a. At the same time,  does not reveal any presence of a precursor in the form of a dilute (2D gas) phase, in which structures smaller than the PEEM resolution are present. In fact,  increases immediately after deposition of a sufficient amount of material to start the third layer, reaches a maximum at 2.5 ML, and decreases until a coverage of 3.0 ML is reached. The coexistence of two emission states is clearly visible in the PEEM images for coverages between 2.2 ML and 2.9 ML: the low emission state corresponds to a condensed third layer and the high emission state represents the closed second layer (possible with a dilute 2D gas in the third layer on top). The spatial homogeneity of the electron yield is restored at 3.0 ML, i.e., at the completion of the third layer.

Since the Anderson method suggests a constant value for the work function for CoPc coverages above 2.0 ML, the changes in the electron yield can no longer be associated with the work function, but rather with the attenuation of the photoelectrons generated at the Ag(100) surface upon passing through the organic thin film. Therefore, we show a different representation of the PEEM data in Figure 4c: we obtain a reference image by averaging three consecutive images with coverage as close as possible to 2.0 ML. Note that the sample is structureless at this growth stage and corresponds to the final state of Δ*W*(Θ). We use this reference image to normalize all subsequent images of the PEEM experiment with coverage above 2.0 ML according to
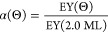
4The quantity α(Θ) derived in this way thus describes the attenuation of the photoelectron yield for Θ ≥ 2 ML. Since the photon energy *h*ν = 4.9 eV is not sufficient to excite electrons from the organic film into the vacuum, all photoelectrons must originate from the silver substrate. If the thickness *d*(Θ) of the film would increase continuously with coverage, the attenuation could be described by the Beer–Lambert law
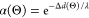
5Here, λ denotes the inelastic mean free path of an electron with energy *E* above the Fermi level and Δ*d*(Θ) = *d*(Θ) – *d*(2 ML). The images of α for selected coverages above 2.0 ML are shown in Figure 4c. At 2.5 ML, the spatial map of the α values clearly reveals a bimodal distribution. The reason is that the sample is locally covered with either 2.0 or 3.0 ML of CoPc. Therefore, coverage-dependent switching of small regions (pixels) from α = 1 (reference layer with a local coverage of 2 ML) to 0.43 (local coverage of 3 ML) is observed, whereas the average value of α(Θ) as well as  in Figure 2a decreases virtually linearly between 2.0 ML and 3.0 ML.

Knowing that the local coverage changes by exactly one monolayer between the images corresponding to 2.0 ML and 3.0 ML in Figure 4c, eq 5 allows us to determine the inelastic mean free path for each pixel via
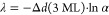
6where Δ*d*(3 ML) = *d*(3 ML) – *d*(2 ML) is the thickness of third CoPc layer on the silver substrate. Averaging over the entire field of view yields λ = Δ*d*(3 ML)·1.18(16). The uncertainty here is an estimate derived from the lateral variation in the two images for 2.0 and 3.0 ML, respectively. Unfortunately, the exact structure and, hence, the thickness of the third ML of CoPc on Ag(100) is not known, but as an estimate, we can take the short axis of the thermodynamically preferred β phase of 0.477 nm for the interlayer spacing of the CoPc film.^39^ This would result in an inelastic mean free path of λ = 0.56(7) nm. However, this is just an upper limit since the molecules in the β phase are slightly tilted. A lower estimate for flat-lying molecules might be the stacking distance between parallel molecules, i.e., 0.34 nm.^40^ This results in λ = 0.40(5) nm.

According to ref (41), the inelastic mean free path λ of electrons in organic media as a function of the electron energy *E* can be expressed as
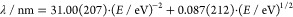
7Assuming that the barrier for photoemission is located at the interface between the vacuum and the organic layer, the maximum kinetic energy of a photoelectron passing through the organic film is given by the energy of the exciting photons *E* = *h*ν = 4.9 eV. The universal curve (given by eq 7) predicts an inelastic mean free path of λ = 1.5(47) nm. Given the large uncertainty of the parameters in eq 7, our value is within the confidence interval. One has to keep in mind that the values reported in ref (41) are average values considering a large number of organic compounds. In ref (42), values are given for specific organic materials, but unfortunately not for phthalocyanines. Nevertheless, compared to the estimate using eq 7, our value is at the lower limit of the confidence interval. The reason could be that we consider only the scattering within a single layer so that boundary effects such as the molecule-vacuum barrier are predominant. When the inelastic mean free path is measured for a thick film, these effects are usually not taken into account.^43^

### 3D Growth

After completion of the third layer, a transition from 2D to 3D growth can be inferred from the PEEM images. The 3D crystallites are imaged darker than the wetting layer and have elongated shapes. Deposition of more material only slightly increases the width or length of the crystallites. Obviously, vertical growth is most favorable. The needles are oriented almost parallel to the step bunches on the surface. The value of  is less than 1, indicating lower electron emission than for the bare Ag(100) surface. In fact, due to the small lateral footprint of the crystallites, most of the surface is covered by the wetting layer, i.e., a three-layer thick CoPc film. In this 3D growth regime,  shows a continuous broadening associated with the coexistence of two emission states on the surface. The negligible emission from the area covered by crystallites corroborates that most photoelectrons originate from the silver surface and have to pass through the organic layer, causing a strong attenuation of the electron emission. After the deposition of an equivalent of 1.5 ML on top of the 3 ML thick wetting layer, the crystallites cover only 3%–5% of the surface. This results in a mean height between 30 ML and 50 ML, assuming the same crystal structure for the wetting layer and the crystallites on top. Based on the inelastic mean free path measured for the third layer of CoPc, such a thickness of the crystallites should completely quench the electron emission from the surface regions covered with crystallites. Yet, we can still detect a small, but finite electron yield from these crystallites. Again, the discrete increase in the layer thickness combined with the limited resolution of the PEEM can provide an explanation: the electron yield measured for a single pixel is a local average of various crystal heights. Since the photoelectron emission is exponentially attenuated, the local spots with the highest emission dominate the arithmetic mean.

## Conclusions

In summary, we have shown that the Fowler–DuBridge photoemission theory allows identifying the main factors responsible for the contrast in PEEM images recorded during in situ growth of ultrathin CoPc films on Ag(100) and using a Hg lamp as the excitation source. Due to the spectral characteristics of the Hg lamp, the photoelectrons originate exclusively from the silver surface. For coverages below 2.0 ML, the electron yield is determined solely by the work function of the sample and the PEEM images directly provide information on the lateral distribution of the work function across the field of view. For coverages above 2.0 ML, the attenuation of the electrons excited in the silver substrate as they pass through the organic layer is the main reason for the decrease in the photoelectron yield. This results in a discrepancy between the work functions extracted from the PEEM data on the basis of the Fowler–DuBridge theory and the values obtained with the Anderson method. Since the layer-by-layer growth continues up to the third layer, we were able to deduce an estimate (0.4 nm–0.6 nm) for the inelastic mean free path of the photoelectrons through the third layer of the CoPc film.

## Supporting Information Available

Details of the Anderson method and the treatment of the PEEM data as well as Fowler–DuBridge transformations using various values for the initial work function (PDF)
