# Supplementary material for: Attenuation of Photoelectron Emission by a Single Organic Layer
Source: ACS Appl Mater Interfaces. 2022 May 10;14(20):23983–9. doi: 10.1021/acsami.2c02996 (PMC9136842; doi:10.1021/acsami.2c02996)
Supplement: Supplementary file 1 — am2c02996_si_001.pdf [file am2c02996_si_001.pdf]

Attenuation of Photoelectron Emission by a Single Organic Layer – Supporting Information –

Thorsten Wagner,[*,†] Grażyna Antczak,[*,‡] Michael Györök,[†] Agata Sabik,[‡] Anna Volokitina,[†] Franciszek Gołek,[‡] and Peter Zeppenfeld[†]

†*Johannes Kepler University, Institute of Experimental Physics, Surface Science Division, Altenberger Str. 69, 4040 Linz, Austria*

‡*University of Wroclaw, Institute of Experimental Physics, Pl. M. Borna 9, 50-204 Wroclaw, Poland*

E-mail: thorsten.wagner@jku.at; grazyna.antczak@uwr.edu.pl

Experimental details

We investigated the growth of ultrathin CoPc films on Ag(100) surfaces in two separate UHV chambers: one hosting the Anderson method,[1,2] the other a photoelectron emission microscope (PEEM). Both vacuum systems reach a base pressure in the lower 10^{-10} mbar range.

The Ag(100) samples were cleaned prior to each deposition experiment by repeated cycles of Ar^+ sputtering (900 V, $\approx 3.8\,\mathrm{A\,cm^{-2}}$ for 30 min) and thermal annealing (660 K for 5 min). After introduction of the CoPc into the vacuum system, the CoPc powders were degassed for about one day. The CoPc molecules were deposited from cells, which were heated resistively to temperatures between 280 °C and 350 °C, depending on the desired deposition rate and

the experimental geometry. In both systems, a similar deposition rate in the range between 0.1 ML/min and 0.3 ML/min was used and the sample was kept at room temperature (RT). Since there was no active temperature control, local heating due to the incident light or the electron beam cannot be excluded. We assume a constant sticking coefficient during deposition to convert the deposition time to a coverage scale. This assumption is supported by the observation of PEEM images, which are homogeneous over the field of view, after equal times in each case – see first column of Fig. S2. We conclude from these homogeneous PEEM images that the first, second and third layers were completelly filled. Following the analysis presented in Ref. 3, the coverage is expressed as an equivalent of the third layer.

Anderson method

With the Anderson method, relative changes of the work function ΔW are measured using an electron beam, which is directed normally towards the Ag(100) surface as described in Refs. 1,2. The cathode of the electron gun is held at +2.5 V and all the other electrodes of the experiment are kept at positive potentials relative to the grounded chamber. Varying the voltage V of the sample from 0 V to 5 V, we acquire the sample current I as function of the voltage V ($I(V)$ curve). The CoPc molecules are deposited onto the surface in a step-wise manner: i) the sample faces the electron gun for $I(V)$ acquisition, ii) then it is rotated towards the CoPc source for the subsequent deposition of a fixed dose. In this way, the set of $I(V)$ curves shown in Fig. S1(a) was collected. Our experiments show that the deposition of CoPc molecules results in changes of two factors: the work function W and the low-energy electron reflectivity R.

Changes in R are evident from the increase of the maximum current (I_{max}) during CoPc deposition. The total current from the electron gun (I_{tot}) is measured by applying a voltage of +180 V to the sample. The electron reflectivity R is calculated according to:

$$R = 1 - \frac{I_{\mathrm{max}}}{I_{\mathrm{tot}}} \tag{S1}$$

The evolution of the electron reflectivity R upon CoPc deposition is shown in Fig. S1(c). The minimum of R is associated with the completion of the first monolayer. The maximum current stays constant during further deposition of CoPc, i.e., for coverages $\Theta \geq 1\,\mathrm{ML}$. This indicates the negligible influence of the thickness of the organic layer on the maximum current extracted from $I(V)$ curves.

To reliably determine the shift between the $I(V)$-characteristics for increasing coverage Θ, one has to compensate for the effect of the changing low-energy electron reflectivity R. This is done by normalizing the current I by the (local) maximum current I_{max}:

$$\widehat{I} = \frac{I}{I_{\mathrm{max}}} \tag{S2}$$

The so normalized $I(V)$-curves are shown in Fig. S1(b). In the next step, the voltages corresponding to a fixed level of $\widehat{I}$ (here $20\,\%$) are determined. The difference to the value for the initial curve (bare Ag(100) surface) then gives the work function shift ΔW. Fig. S1(d) shows a complete series of the work function changes $\Delta W(\Theta)$ obtained with Anderson method during deposition of CoPc on a Ag(100) surface held at room temperature. The amplitude and the shape of the $\Delta W(\Theta)$ curve were reproduced in four experiments (not shown here), in which the deposition rates varied from $0.1\,\mathrm{ML/min}$ to $0.3\,\mathrm{ML/min}$.[1,2] All curves reach a minimum value of about $-0.3\,\mathrm{eV}$ and saturate for $\Theta \geq 2\,\mathrm{ML}$ at about $-0.05\,\mathrm{eV}$.

PEEM measurements

The experiments were carried out with a commercial PEEM from Focus with integrated sample stage. For the photoelectron excitation, a high-pressure Hg lamp was used, which has a strong emission line at $\lambda = 253\,\mathrm{nm}$ (corresponding to $h\nu = 4.9\,\mathrm{eV}$).[4] The molecular source and the lamp were mounted at an angle of 65° with respect to the surface normal. A commercial evaporator (ventiotec OVD3) with quartz crucibles was used to deposit the

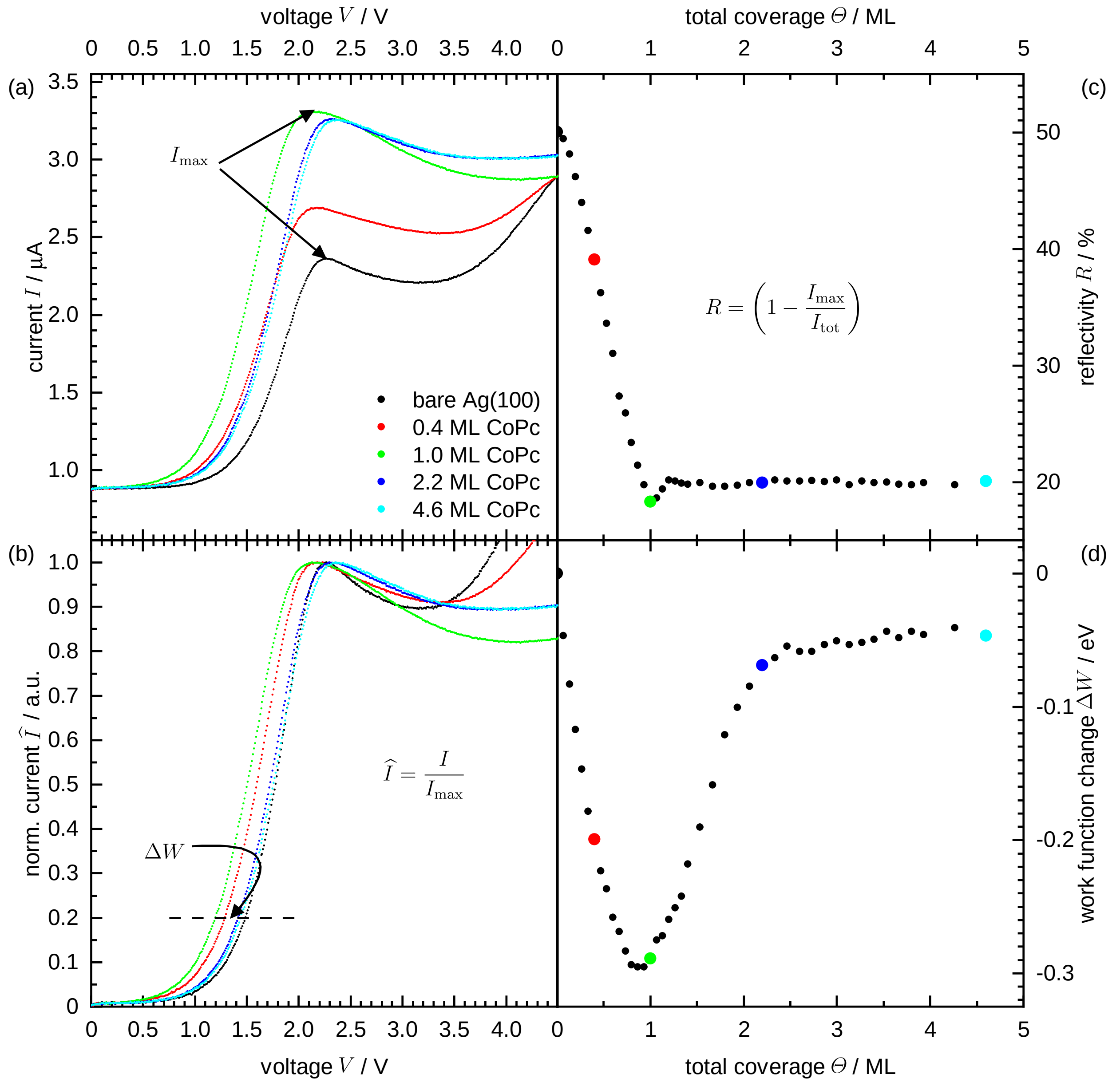

Figure S1: CoPc deposition on Ag(100) at room temperature investigated with the Anderson method: (a) $I(V)$ curves acquired after incremental deposition of CoPc, (b) same data as in (a) but normalized by the local maximum current I_{max}, (c) changes in the low-energy electron reflectivity R and (d) changes of the work function during deposition of CoPc. The respective data points extracted from the curves shown in (a) and (b) are highlighted in (c) and (d) with the corresponding colors and larger symbols.

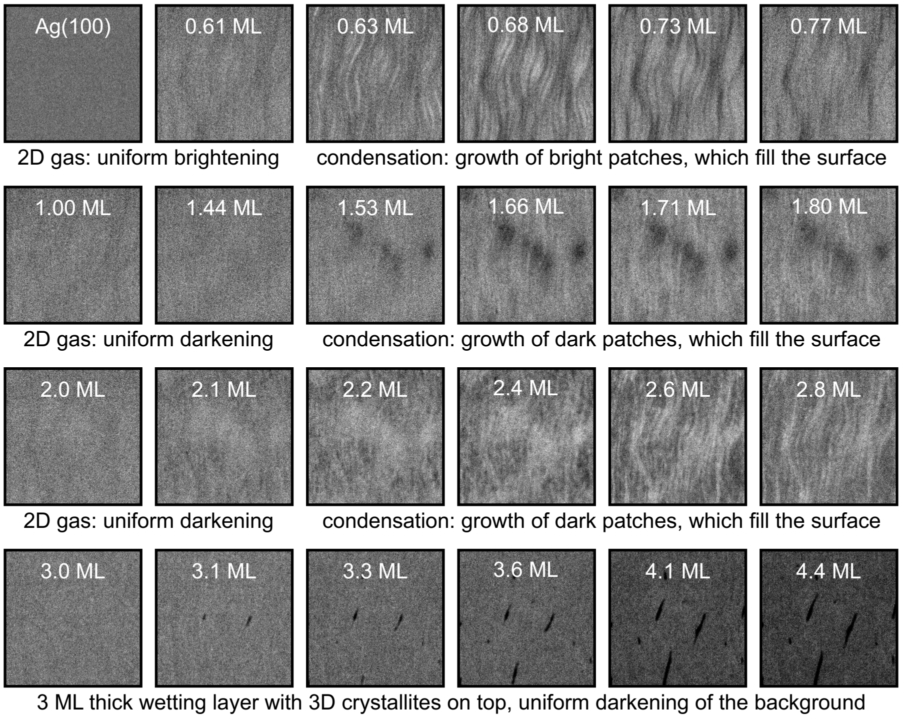

Figure S2: Series of PEEM images with optimized contrast for each image recorded during deposition of CoPc molecules on a Ag(100) surface. The images represent an area on the surface with dimensions 60 µm × 60 µm.

molecules. The deposition of CoPc can be executed in a continuous or in a step-wise manner using a shutter to block the flux of the molecules at any given time. The PEEM images are recorded continuously at a frame rate of 0.5 frames/s with a field of view of about 130 µm. The deposition rates used in the PEEM and Anderson experiments were similar. The actual experiment shown in this publication was carried out in a step-wise manner to match the conditions of the Anderson experiment: The shutter of the molecular source was opened for 25 frames (corresponding to a time interval of 50 s) and then closed for the same time interval. The mean electron yield (MEY) across the field of view of 130 µm obtained with or without intermittent interruption are almost identical confirming negligible changes while the shutter was closed. Just upon the onset of 3D growth, the distribution of the electron yield across the image and the corresponding mean value MEY show tiny jumps between subsequent deposition steps (see Fig. 2 of the main paper). This indicates that the 3D crystallites and the 2D molecular gas on top of the wetting layer are not totally in thermal

equilibrium as long as molecules are deposited. We assume that the density of the 2D gas phase is reduced when the shutter of the source is closed, because molecules are incorporated into the 3D islands. Fig. S2 show a series of PEEM images obtained during deposition of CoPc on a Ag(100) surface. The images were corrected for the dark image D (taken without illumination) and the background B (contribution of multichannel plate, inhomogeneous illumination, apertures, etc) to obtain the local electron yield $EY(x, y)$ via

$$EY(x, y) \propto \frac{I(x, y) - D(x, y)}{B(x, y) - D(x, y)} \tag{S3}$$

Here $I(x, y)$ is the intensity in an image pixel at position (x, y). For image processing the software distribution Fiji was used.[5] Details about the interpretation of the normalized standard deviation $\widehat{SD}(\Theta)$ shown in Fig. 3 of the main paper and a data set with a continuous deposition of CoPc molecules on a Ag(100) surface can be found in Ref. 3.

Fowler-DuBridge

The conversion between work function changes ΔW measured with the Anderson method and those extracted from the mean electron yield MEY is based on the Fowler-DuBridge theory.[6–8] A central parameter for this conversion is the photon energy (here $h\nu = 4.9\,\mathrm{eV}$). Since the Anderson method does not provide the absolute value of the work function of the bare substrate W_0 or the thin film, the initial work function is an additional parameter, which has to be determined independently. In Fig. 2 of the main paper, we used $W_0 = 4.64\,\mathrm{eV}$ for the work function of the bare Ag(100) substrate surface in agreement with Ref. 9.

Fig. S3 shows the same photoelectron emission data but using values for the initial work function W_0 between 4.54 eV and 4.74 eV. For coverages up to 2.0 ML, a fairly good match of the amplitude and overall shape between the measured Anderson data and the converted PEEM data is obtained for $W_0 = 4.64\,\mathrm{eV}$.

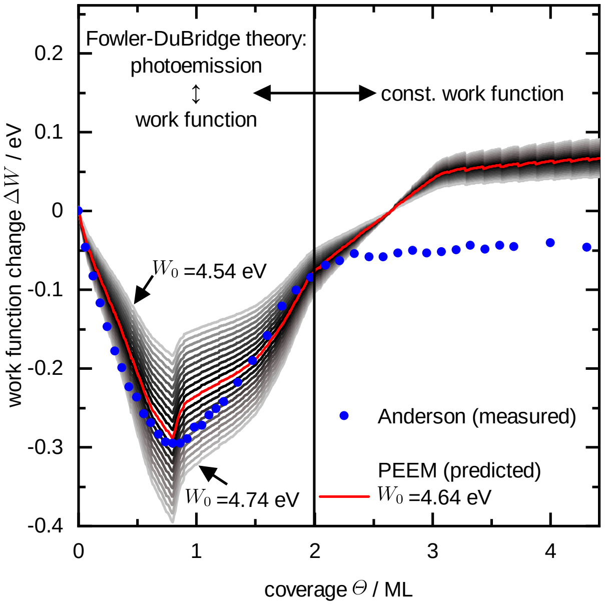

Figure S3: The blue dots show the work function changes upon deposition of CoPc onto a Ag(100) surface as measured with the Anderson method. These data are compared with work function changes derived from the transient of the mean electron yield (MEY) measured in PEEM and subsequent conversion using the Fowler DuBridge theory. The curves were calculated for various values of the initial work function W_0 between 4.54 eV and 4.74 eV. The best match between the Anderson data and the PEEM ones is found by eye for an initial work function of 4.64 eV.

References

(1) Sabik, A.; Gołek, F.; Antczak, G. Note: Work Function Change Measurement via Improved Anderson Method. *Review of Scientific Instruments* **2015**, *86*, 056111.

(2) Sabik, A.; Gołek, F.; Antczak, G. Reduction of the Low-Energy Electron Reflectivity of Ag(100) upon Adsorption of Cobalt Phthalocyanine Molecules. *The Journal of Physical Chemistry C* **2016**, *120*, 396–401.

(3) Wagner, T.; Antczak, G.; Ghanbari, E.; Navarro-Quezada, A.; Györök, M.; Volokitina, A.; Marschner, F.; Zeppenfeld, P. Standard Deviation of Microscopy Images used as Indicator for Growth Stages. *Ultramicroscopy* **2022**, *233*, 113427.

(4) Wagner, T.; Ghanbari, E.; Huber, D.; Zeppenfeld, P. The Growth of α-Sexithiophene

Films on Ag(111) Studied by means of PEEM with Linearly Polarized Light. *Ultramicroscopy* **2015**, *159*, 464–469.

(5) Schindelin, J. et al. Fiji: an Open-Source Platform for Biological-Image Analysis. *Nature Methods* **2012**, *9*, 676–682.

(6) DuBridge, L. A. A Further Experimental Test of Fowler's Theory of Photoelectric Emission. *Phys. Rev.* **1932**, *39*, 108–118.

(7) DuBridge, L. A. Theory of the Energy Distribution of Photoelectrons. *Phys. Rev.* **1933**, *43*, 727–741.

(8) Fowler, R. H. The Analysis of Photoelectric Sensitivity Curves for Clean Metals at Various Temperatures. *Phys. Rev.* **1931**, *38*, 45–56.

(9) Michaelson, H. B. The Work Function of the Elements and its Periodicity. *Journal of Applied Physics* **1977**, *48*, 4729–4733.
